# Subacute Onset of Perineural Rhino-Cerebral Mucormycosis Mimicking Giant Cell Arteritis: A Case Report

**DOI:** 10.7759/cureus.112473

**Published:** 2026-07-11

**Authors:** Ravi Ambati, David Prentice, Andrew Thompson, Chris Heath

**Affiliations:** 1 Department of Neurology, Royal Perth Hospital, Perth, AUS; 2 Department of Neurosciences, Perron Institute for Neurological and Translational Science, Perth, AUS; 3 Neurological Intervention and Imaging Service of Western Australia (NIISwa), Royal Perth and Sir Charles Gairdner Hospitals, Perth, AUS; 4 Department of Microbiology, PathWest Laboratory Medicine, Fiona Stanley Hospital, Perth, AUS; 5 Department of Infectious Diseases, Fiona Stanley Hospital, Perth, AUS; 6 School of Medicine, University of Western Australia, Perth, AUS

**Keywords:** greater palatine nerve, greater superficial petrosal nerve, neuralgia, pterygopalatine fossa, pterygopalatine ganglion, rhino-cerebral mucormycosis, vidian nerve

## Abstract

A diabetic woman in her 40s presented with a subacute onset of a left temporal headache with no other symptoms, especially no visual or nasal symptoms. An initial diagnosis of giant cell arteritis was made due to a raised erythrocyte sedimentation rate (ESR) and the presence of jaw claudication; however, the temporal artery biopsy was negative. Corticosteroid therapy was commenced, but subsequent computed tomography (CT) imaging of the face revealed extensive left maxillary sinus disease with bony erosion of the greater palatine and vidian nerve canals. The ear, nose, and throat (ENT) examination showed no mucosal necrosis, and initial biopsies were negative for mucormycosis. Given the imaging findings, a tentative diagnosis of mucormycosis was made, and the patient was treated with liposomal amphotericin B. Repeat deep biopsies confirmed the diagnosis of mucormycosis. The nature of the headache was consistent with pterygopalatine neuralgia, given the headache characteristics, taste abnormality, and magnetic resonance imaging (MRI) findings at the pterygopalatine fossa. The importance of the pterygopalatine fossa as a conduit for potential mucormycosis perineural spread is highlighted. This case also underscores mucormycosis as an important mimic of giant cell arteritis, particularly in patients presenting with new-onset unilateral headache and cranial neuropathies.

## Introduction

Rhino-cerebral mucormycosis (RCM) is a life-threatening invasive fungal infection that predominantly affects immunocompromised patients and those with poorly controlled diabetes mellitus. The classical acute presentation features rapidly progressive nasal and palatal necrosis, orbital involvement, and fulminant cranial nerve deficits. However, chronic or indolent forms of RCM are increasingly recognised, presenting with non-specific symptoms such as headache and facial pain that may mimic more common conditions, including bacterial sinusitis and giant cell arteritis (GCA) [1-3].

While angioinvasion is the hallmark of mucormycosis pathogenesis, accumulating evidence supports perineural spread as an additional mechanism of disease dissemination, particularly along branches of the trigeminal nerve [4,5]. The pterygopalatine fossa, a critical anatomical crossroads containing the sphenopalatine (pterygopalatine) ganglion, vidian nerve, and palatine nerves, represents a key potential conduit for such perineural extension [1].

We present a case of subacute RCM in a poorly controlled diabetic patient who initially presented with unilateral headache and jaw claudication, mimicking GCA. The clinical presentation was consistent with sphenopalatine (pterygopalatine) neuralgia, and imaging demonstrated perineural spread along the vidian and palatine nerves.

## Case presentation

A 45-year-old Indigenous Australian woman from remote rural Western Australia presented with a three-week history of left-sided otalgia, progressive frontotemporal headache, and jaw claudication. The headache was constant, deep, and dull, radiating to the left temporal region and worsening with mastication. Over several days, she developed numbness involving her left cheek in an infraorbital (V2) nerve distribution, altered taste, and mild left soft-palate numbness. She denied visual changes, rhinorrhoea, limb neurology, or constitutional features at presentation.

Her past medical history included poorly controlled type 2 diabetes mellitus (HbA1c 11.3%), hypertension, hyperlipidaemia, and recurrent sinusitis. She had a longstanding difficulty adhering to metformin. She was a non-smoker, consumed minimal alcohol, and frequently engaged in gardening.

She was initially managed in a regional hospital, where bacterial sinusitis was suspected, and oral cefalexin was prescribed without clinical improvement. Persistent erythrocyte sedimentation rate (ESR) elevation, jaw claudication, and new-onset temporal headache led to a provisional diagnosis of temporal arteritis, and high-dose prednisolone (50 mg daily) was started. Persistent pain and sensory changes prompted transfer to a tertiary hospital.

On examination, there was sensory loss in the left maxillary (V2) nerve distribution as well as reduced taste sensation on the left side of her tongue. There were no motor deficits, visual disturbances, or other cranial nerve abnormalities. Ophthalmology review was unremarkable, and the ear, nose, and throat (ENT) examination revealed no signs of acute sinusitis.

Investigations

**Figure 1:**
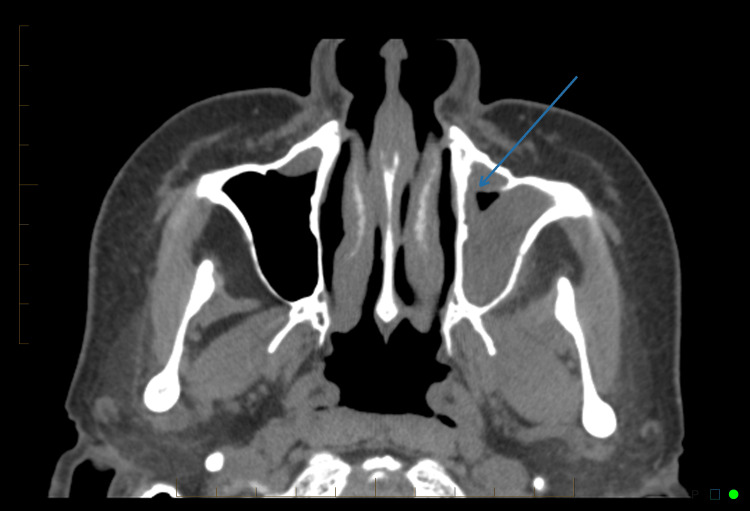
Axial non-contrast CT of the paranasal sinuses demonstrating asymmetric mucosal thickening of the left maxillary sinus (blue arrow), more marked than the contralateral side, without evidence of bony destruction CT: computed tomography

Non-contrast computed tomography (CT) of the head showed bilateral maxillary antral mucosal thickening, more marked on the left, but no bone destruction or intracranial abnormality (Figure 1).

Temporal artery ultrasound showed no features suggestive of temporal arteritis.

**Figure 2:**
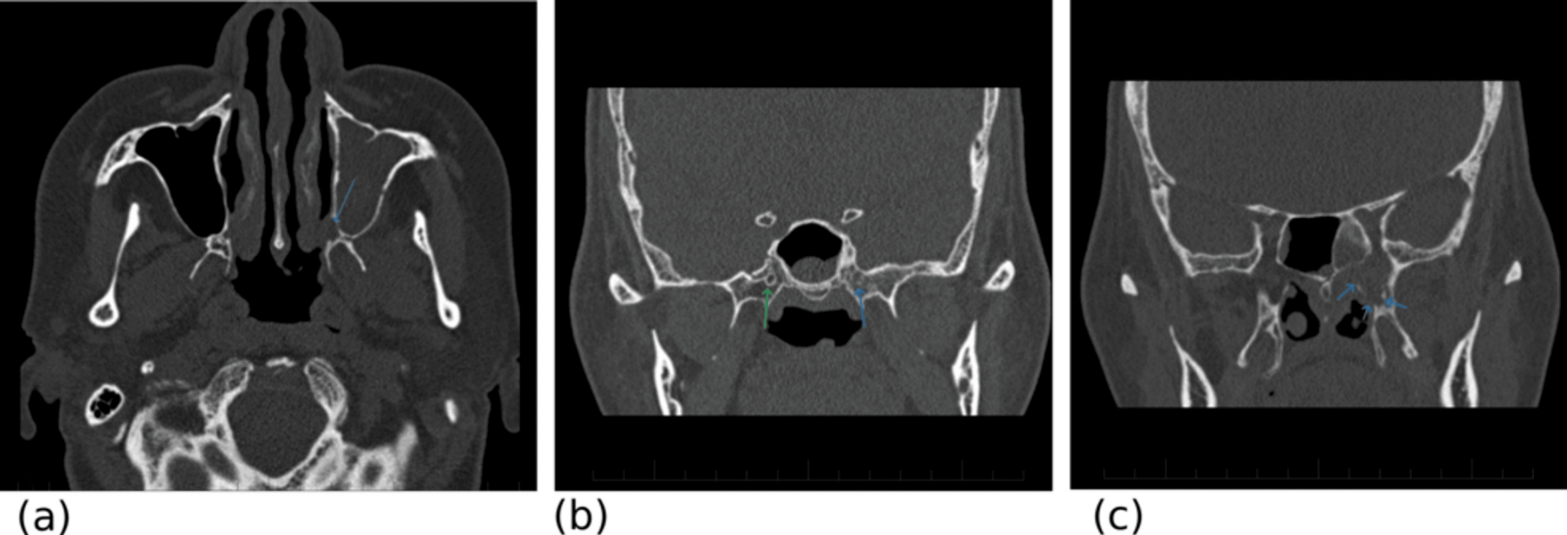
CT imaging demonstrating bony erosion consistent with perineural mucormycosis. (a) Axial CT showing widening of the left greater palatine canal (arrow), indicating early involvement of the greater palatine nerve. (b) Coronal CT demonstrating erosion of the anterior margin of the left vidian canal (blue arrow) with a normal right vidian canal (green arrow) for comparison. (c) Coronal CT showing erosion of the margins of the expanded left pterygopalatine fossa, the epicentre of the fungal perineural disease (arrows) CT: computed tomography

Repeat CT of the face confirmed interval progression, with confluent soft tissue infiltration in the retromaxillary region and osteolysis of the vidian canal, the pterygoid process of the sphenoid bone, and the greater and lesser palatine canals (Figure 2).

**Figure 3:**
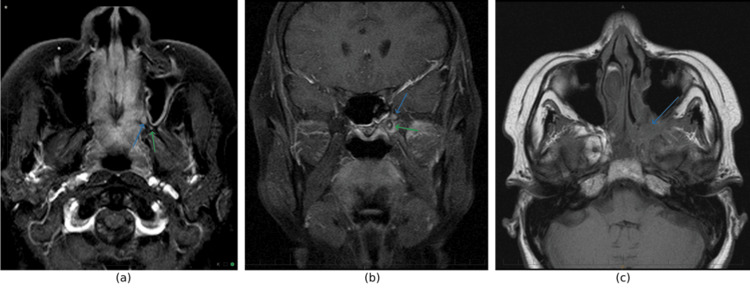
MRI demonstrating perineural spread of mucormycosis. (a) Axial post-gadolinium T1-weighted MRI demonstrating enlargement and enhancement of the greater palatine nerve (blue arrow) and lesser palatine nerve (green arrow). (b) Coronal T1-weighted post-contrast fat-saturated MRI demonstrating enlargement and enhancement of V2 within the foramen rotundum (blue arrow) and of the vidian nerve (green arrow), with abnormal enhancing tissue replacing the normal fat in the left pterygopalatine fossa, consistent with perineural spread. (c) Axial T1-weighted MRI demonstrating preserved normal fat in the right pterygopalatine fossa and pterygomaxillary fissure, in contrast to expansile intermediate-signal tissue replacing the fat within the left pterygopalatine fossa, in keeping with infiltrative fungal disease (arrow) MRI: magnetic resonance imaging

Magnetic resonance imaging (MRI) of the head and sinuses demonstrated an inflammatory process involving the left sphenopalatine foramen, pterygopalatine fossa, and infratemporal fossa, with enhancement of the greater and lesser palatine nerves, the vidian nerve, and the maxillary nerve, consistent with perineural spread (Figure 3). 

**Figure 4:**
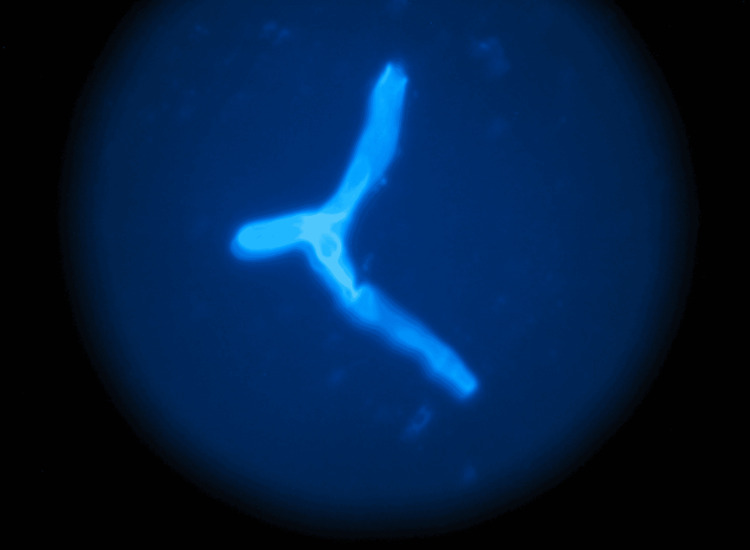
Calcofluor white-stained smear demonstrating broad, aseptate fungal hyphae consistent with mucormycosis (×400 magnification)

An initial biopsy of the left maxillary sinus and uncinate process was non-diagnostic. A repeat deep biopsy from the left sphenoid and pterygopalatine region revealed adjacent tissue necrosis with broad, non-septate hyphae on calcofluor-stained smears (Figure 4), confirming the diagnosis of mucormycosis.

Treatment

**Figure 5:**
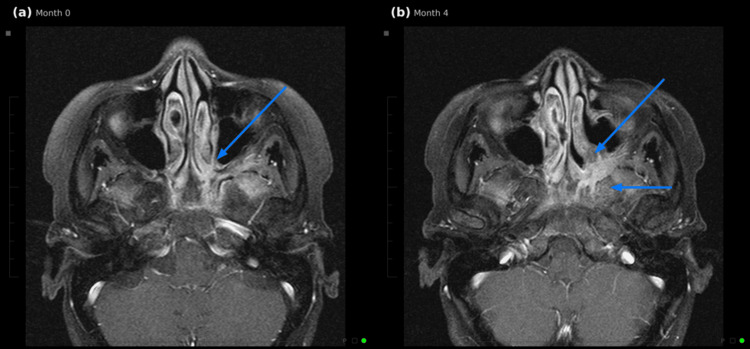
Axial post-gadolinium T1-weighted fat-saturated MRI demonstrating interval progression of perineural mucormycosis. (a) Month 0: Abnormal enhancing tissue within the left pterygopalatine fossa and retromaxillary region (arrow), consistent with infiltrative fungal disease. (b) Month 4: Interval progression with increased infiltration of the retromaxillary fat and medial and lateral pterygoid muscles (arrows), with persistent enhancement along the course of the vidian and maxillary nerves MRI: magnetic resonance imaging

Corticosteroids were discontinued, and intravenous liposomal amphotericin B at 5 mg/kg/day was commenced and continued for four months. Repeat MRI initially demonstrated interval progression, with increased infiltration of the retromaxillary fat and medial and lateral pterygoid muscles and persistent enhancement of the vidian and maxillary nerves (Figure 5). Oral posaconazole was added as adjunctive therapy and subsequently continued as maintenance monotherapy for another four months. 

Outcome and follow-up

**Figure 6:**
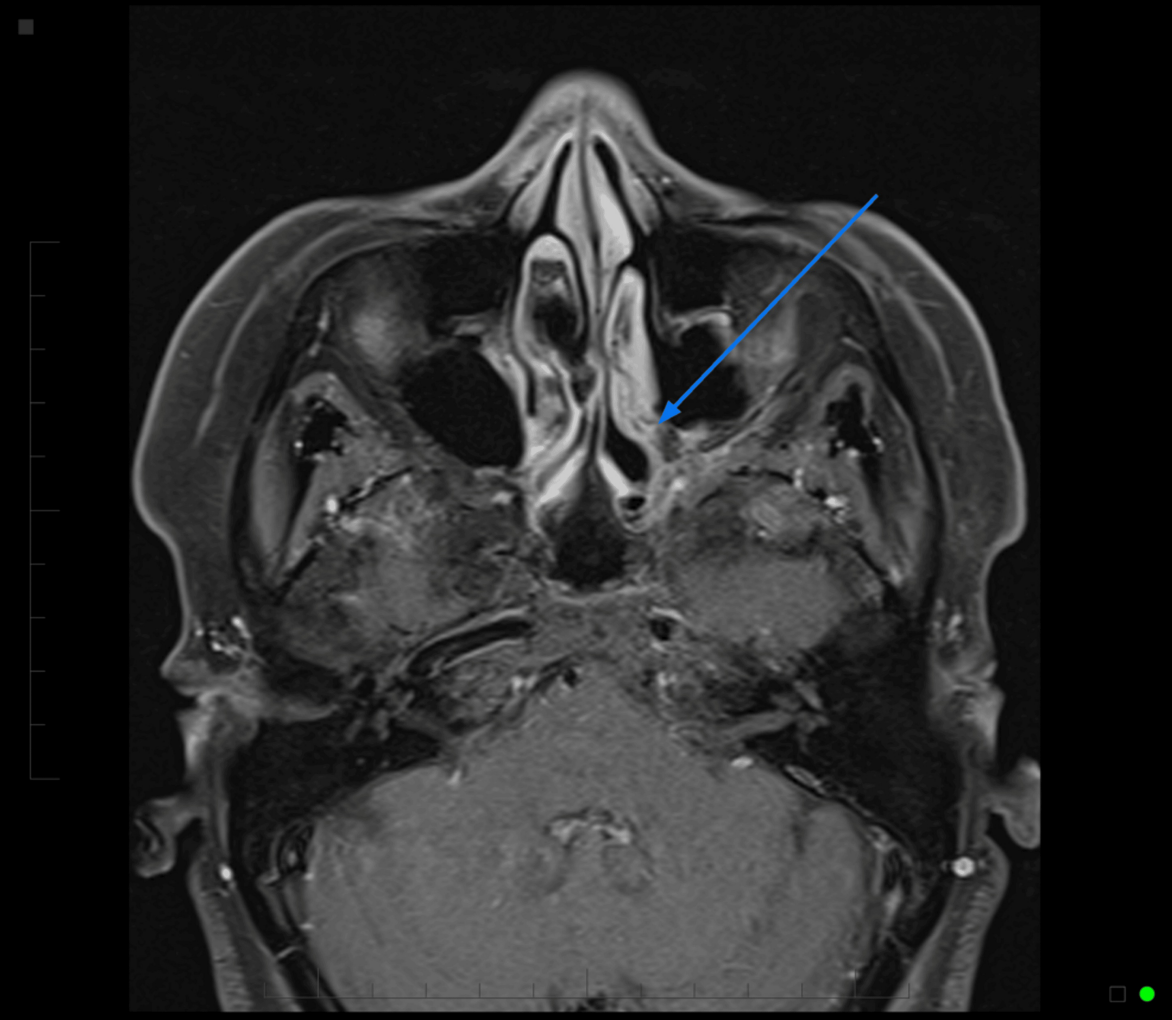
Axial post-gadolinium T1-weighted fat-saturated MRI following antifungal therapy demonstrating improvement with resolution of abnormal enhancement in the retromaxillary region and vidian canal. Residual soft tissue thickening within the left pterygopalatine fossa persists (arrow) MRI: magnetic resonance imaging

Surgical debridement was considered but deemed impracticable due to skull base involvement. Following more than four months of liposomal amphotericin B therapy, MRI ultimately demonstrated improvement in the pterygopalatine fossa and vidian canal, although soft tissue changes in the pterygopalatine region remained (Figure 6). Clinically, the patient continued to experience left V2 sensory loss and neuropathic pain, although there was no further progression. Amphotericin B was discontinued after an infectious disease team review, and she remained on maintenance oral posaconazole. The duration of posaconazole therapy was for another four months, with variable adherence. 

At the long-term follow-up, she continues to have a stable sensory deficit in the left maxillary (V2) nerve distribution.

## Discussion

Sphenopalatine neuralgia

Sphenopalatine neuralgia (Sluder's neuralgia) is a headache syndrome characterised by severe unilateral, boring or burning pain around the eye, radiating to the maxilla, zygoma, and mastoid. The pain is typically continuous and associated with unilateral reduced lacrimation and altered taste sensation involving the hard and soft palate, mediated by the greater and lesser palatine nerves [1].

Although our patient did not fulfil all classical diagnostic criteria, we believe this syndrome best explains her pain, given the absence of nasal symptoms and lack of mucosal abnormalities on inspection or biopsy. Furthermore, CT imaging demonstrated erosion of the bony canals of the palatine nerves, supporting involvement of this pathway.

The sphenopalatine ganglion contains both somatic and autonomic nerve fibres. Its autonomic supply includes innervation to the ipsilateral lacrimal gland and to the palatal taste buds and mucous glands via the greater petrosal nerve (GPN). The GPN attracted interest during the COVID-19 pandemic, where several cases of RCM were reported presenting with facial nerve palsy. In some of these cases, Schirmer's testing demonstrated reduced ipsilateral tear production, further supporting this proposed mechanism of pathogenesis [2].

Perineural spread

Mucormycosis typically spreads via the vascular system, causing tissue ischaemia and infarction, which accounts for the characteristic black necrotic mucosal lesions. More recently, increasing attention has been given to the possibility of perineural spread via the endoneurium and perineurium of peripheral and cranial nerves [4,5]. Histopathological evidence of this mechanism has been demonstrated in both autopsy specimens and surgically obtained tissue [4,5].

MRI features suggestive of perineural spread include nerve thickening and expansion, T2-weighted hyperintensity, and enhancement on post-gadolinium T1-weighted sequences. Although perineural extension is a recognised pathway for RCM dissemination, our case is notable for demonstrating radiological involvement of both the vidian and palatine nerves.

Chronic mucormycosis

Chronic or indolent RCM is defined as pathologically confirmed sinus mucormycosis persisting for more than one month [3]. Clinically, it often presents with headache or facial pain associated with single-sinus disease and can therefore be easily misdiagnosed as bacterial sinusitis. Chronic RCM has been reported in both immunocompetent and immunosuppressed patients, although most "immunocompetent" cases have underlying diabetes or are newly diagnosed diabetics. Gutierréz-Delgado et al. reviewed 22 cases of chronic RCM; 25% were immunocompetent, the diagnosis was made by biopsy, and cases had an 85% survival rate [6].

The increasing prescription of statins has been proposed as a possible contributor to the lower incidence of mucormycosis in high-income countries. Petrikkos et al. speculated that statins may exert inhibitory effects on fungal growth, potentially accounting for the relatively low rates of rhino-orbital-cerebral mucormycosis reported in Western countries compared with under-resourced regions such as India [7]. Our patient was not taking a statin prior to the onset of infection.

GCA mimics

RCM can mimic the constellation of symptoms of GCA, as both diseases cause vascular ischaemia. GCA can result in scalp and tongue necrosis due to the occlusion of the branches of the external carotid artery. Jaw claudication is a key symptom of GCA, thought to be due to the involvement of the maxillary artery, which can also occur in RCM, as in our case. A case report of maxillary artery involvement in GCA has been shown on fluorodeoxyglucose (FDG)-positron emission tomography (PET) [8]. The maxillary artery also supplies arterial branches to the sphenopalatine ganglion, allowing us to speculate that ischaemia to this nerve may contribute to the headache symptomatology of both conditions.

The headache pattern in GCA has been poorly documented. Vincenten and Mulleners conclude that the commonest pattern is a continuous unilateral temporal headache with nausea, jaw claudication, amaurosis fugax, and diplopia occurring in 30% of patients [9]. The differential diagnosis of GCA is broad and includes vasculitides (antineutrophil cytoplasmic antibody (ANCA)-associated vasculitis, polyarteritis nodosa, calciphylaxis, and IgG4-related disease), infections (varicella, syphilis, aspergillosis, and tuberculous meningitis), malignancies (amyloidosis and lymphoma), migraine, and temporomandibular disorders.

A number of cases of suspected GCA diagnosed with sudden loss of vision and treated with steroids subsequently developed RCM. Hawkins et al. reported a 72-year-old diabetic with optic perineuritis treated with steroids who developed RCM; they reviewed six similar cases of RCM mimicking GCA [10]. Warning signs in these patients include negative inflammatory markers, the presence of sinus disease, diabetes, and neuropathic pain. Careful history and examination in suspected RCM may reveal sphenopalatine dysfunction with altered taste and/or lacrimation.

Key learning points

This case illustrates several important clinical lessons. First, RCM can closely mimic GCA, presenting with headache, facial pain, and cranial neuropathies, particularly in patients with poorly controlled diabetes. Clinicians should maintain a high index of suspicion for RCM when temporal arteritis is suspected in a diabetic patient, especially when temporal artery biopsy is negative or inflammatory markers are atypical. Second, the sphenopalatine ganglion represents an important and under-recognised anatomical route for the perineural spread of mucormycosis to the skull base, and involvement of this structure may explain the characteristic headache pattern and taste disturbance seen in some cases. Third, indolent or subacute forms of RCM can occur and may present without the classical necrotic mucosal lesions or fulminant features typically associated with this infection, making early clinical recognition challenging. Finally, RCM can occur not only in immunocompromised hosts but also in immunocompetent individuals and in diabetics without ketoacidosis, broadening the population at risk beyond the traditionally recognised high-risk groups.

## Conclusions

Our case highlights the diagnostic challenge of subacute RCM presenting with sphenopalatine neuralgia and initially mistaken for GCA in a poorly controlled diabetic. Misdiagnosis of GCA may occur when age and the presence of sinus disease are overlooked. Diagnosis of RCM can be difficult and typically requires a high index of suspicion, detailed imaging assessment, and often multiple deep biopsies for confirmation. Early recognition and antifungal treatment are critical to prevent death and irreversible neurological injury and to improve outcomes.
